# Effect of Polymer/Nano-Clay Coatings on the Performance of Concrete with High-Content Supplementary Cementitious Materials under Harsh Exposures

**DOI:** 10.3390/ma17051030

**Published:** 2024-02-23

**Authors:** M. A. Abuzeid, M. T. Bassuoni, M. R. Sakr

**Affiliations:** 1 Department of Civil Engineering, University of Manitoba, Winnipeg, MB R3T 5V6, Canada; abuzeidm@myumanitoba.ca; 2 Department of Civil Engineering, Faculty of Engineering at Shoubra, Benha University, Cairo 11629, Egypt; mohamed.ramadan@feng.bu.edu.eg

**Keywords:** surface coatings, nanocomposites, concrete, physical salt attack, salt–frost scaling, durability

## Abstract

In recent concrete research, a novel category of coatings has emerged: polymers/nanoparticles blends. The efficacy of such coatings warrants extensive examination across various concrete mixtures, particularly those incorporating high-volume supplementary cementitious materials (SCMs) to mitigate carbon footprints, an industry imperative. This study used three vulnerable concrete mixtures to assess the effectiveness of ethyl silicate and high-molecular-weight methyl methacrylate blended with 2.5% and 5% halloysite and montmorillonite nano-clay. Findings from physical, thermal, and microstructural analyses confirmed vulnerabilities in concretes with a high water-to-binder ratio (0.6) under severe exposure conditions, notably with high SCM content (40% and 60% fly ash and slag, respectively). Neat ethyl silicate or high-molecular-weight methyl methacrylate coatings inadequately protected those concretes against physical salt attacks and salt–frost scaling exposures. However, the incorporation of halloysite nano-clay or montmorillonite nano-clay in these polymers yielded moderate-to-superior concrete protection compared to neat coatings. Ethyl silicate-based nanocomposites provided full protection, achieving up to 100% improvement (no or limited surface scaling) against both exposures, particularly when incorporating halloysite-based nano-clay at a 2.5% dosage by mass. In contrast, high-molecular-weight methyl methacrylate-based nano-clay composites effectively mitigated physical salt attacks but exhibited insufficient protection throughout the entire salt–frost scaling exposure, peeling off at 15 cycles.

## 1. Introduction

Reinforced concrete structures are expected to meet their intended service life, typically ranging from 75 to 120 years according to ACI 365.1R-17 [1]. However, the concrete of certain infrastructures may deteriorate prematurely, compromising their functionality due to harsh single or concurrent exposures [2,3]. Consequently, substantial portions of government budgets are allocated to the repair, rehabilitation, and sometimes reconstruction of infrastructural facilities instead of expanding asset networks [4]. Physical salt attacks (PSAs) and salt–frost scaling are challenging conditions that concrete may encounter during service. A PSA is a form of wetting/drying (W/D) damage mechanism prevalent in hot, arid conditions with salt-rich environments. The formation of crystallized salt within the near-surface pores of concrete induces elevated tensile stresses (10–20 MPa), resulting in the scaling and detachment of the surface layer [5,6]. Salt–frost scaling, on the other hand, poses significant challenges for transportation agencies in cold climates as a result of using de-icing chemicals on concrete flatwork (e.g., sidewalks, driveways, pavements) [7]. The combination of deicers and freezing/thawing cycles can lead to severe scaling and cracking on the concrete surface, disrupting essential transportation functions and incurring substantial rehabilitation costs [8].

Enhancing the surface layer of concrete by coatings has emerged as a valuable protective technique, offering a cost-effective alternative to achieve sufficient protection instead of resorting to expensive strategies, such as high-performance concrete [3,9]. Commercial coatings are employed directly based on the recommendations of their manufacturers, falling into four categories: (a) coatings forming an isolating membrane (e.g., epoxy); (b) hydrophobic agents (e.g., silane); (c) surface pore blockers (e.g., sodium silicate); and (d) multifunctional treatments (e.g., ethyl silicate) [10]. Coatings exhibit varying degrees of effectiveness in protecting concrete depending on the specific circumstances, yet they may prove ineffective under severe conditions. For instance, Suleiman et al. [11] determined that specific coatings, such as silane, were effective against mild physical salt attack (PSA) conditions regardless of the mixture. However, Sakr et al. [12] observed that silane was unsuccessful in providing protection under more severe and aggregated PSA conditions, which suggests that special coatings may be necessary to protect concrete under such harsh conditions.

Few endeavors explored innovative surface treatments leveraging nanotechnology aimed at safeguarding concrete from harsh conditions (e.g., [13,14,15,16,17,18,19,20,21,22,23,24,25,26]). These initiatives involve investigating nanoparticle solutions as standalone treatments and improving existing commercially available coatings with nanoparticles. Hence, a new class of nano-based coatings, the suspension of nanoparticles (at least one dimension is less than 100 nm) into polymer resins or water, recently emerged in concrete research. For instance, Leung et al. [13] observed that incorporating 1–5% nano-clay with epoxy or silane improved the barrier performance of concrete, as shown by tests measuring water vapor transmission and salt spray. Woo et al. [19] found that the nano-clay blended silane coating effectively filled micro-pores on concrete surfaces, leading to a reduction in the percolation of liquids and gases. Guo et al. [20] developed epoxy resin coatings reinforced with TiO_2_-graphene at dosages ranging from 0.3 to 1.0 wt%, demonstrating enhanced impermeability compared to neat epoxy resin coatings. Li et al. [21,22,23] investigated the impact of adding nano-SiO_2_ at dosages of 0.5, 1.5, and 3.0 wt% to polyurethane, epoxy resin, and chlorinated rubber paints applied on concrete. Their findings indicated that the inclusion of nano-SiO_2_ particles reduced the damage caused by ultraviolet rays to polymer molecules and improved concrete’s long-term resistance to chloride ion attacks and carbonation. In another study by Dorado et al. [24], it was reported that 3 wt% Fe_2_O_3_ nanoparticles were incorporated into a two-part epoxy resin coating. Concrete slabs with the modified coating underwent accelerated ageing (chemical corrosion and surface abrasion) showed significantly improved performance compared to unmodified coatings. 

Sakr and Bassuoni [25] explored water-based nano-silica solutions and silane-based nanocomposites for concrete subjected to PSA and salt–frost scaling. The study identified the 50% dosage of water-based nano-silica solutions and 5% by mass of nano-clay particles with silane as effective measures for superior protection against both exposures. In addition, in another study by the authors [26], incorporating nanoparticles into silane at 5% dosage offered sufficient protection in various exposures, with nano-clay particles more effective than nano-silica, while the 10% loading ratio showed inadequate performance due to particle agglomeration. Methyl methacrylate-based nanocomposites were only effective against PSA exposure and failed in salt–frost scaling conditions [26]. However, these studies were applied on plain concrete mixtures. 

### Research Significance

Currently, there remains a scarcity of data regarding the development and testing of diverse nanocomposites utilizing various base resins against rigorous durability conditions. Additionally, there has been a notable absence of research focused on safeguarding concrete containing high levels of supplementary cementitious materials (SCMs) when subjected to severe conditions, such as wetting and drying cycles with salt-rich solutions and freezing and thawing cycles accompanied by de-icing salts (e.g., on flatwork in cold regions). As outlined in the introduction, prior studies [13,14,15,16,17,18,19,20,21,22,23,24,25,26] have highlighted the promising functionality of nanocomposites as viable strategies for enhancing common polymeric coatings. However, these investigations solely evaluated the effectiveness of nanocomposites on concrete prepared with plain cement, underscoring the need for further validation with other types of concrete, including those with blended binders.

Therefore, the motivation and contribution of the present study lie in assessing the capability of ethyl silicate and high-molecular-weight methacrylate (HM) mixed with nano-clay particles to protect the concrete-comprising high content of SCMs (fly ash and slag) under harsh conditions. The key findings from this study are anticipated to offer insights for safeguarding and rehabilitating elements containing high-volume SCMs facing durability challenges. Moreover, they have the potential to contribute to the refinement of guides, codes, and specifications for the development of durable concrete capable of withstanding PSA and salt–frost scaling conditions.

## 2. Materials and Experiments

### 2.1. Materials

#### 2.1.1. Concrete Mixtures

Three concrete mixtures were employed in this study, maintaining a constant binder content of 400 kg/m^3^. Mixtures were designed to implement single and binary cementitious binders at a fixed *w*/*b* of 0.60 to represent various types of low-quality concrete in need for protection, which is typical for residential construction in North America. General use (GU) Portland cement, Class F fly ash (FA), and Grade 100 slag (SG), which conform to the specifications of CAN/CSA-A3001 [27], constituted the primary components of the binders. Single-binder concrete comprised 100% GU cement, whereas binary-binder mixtures involved replacement of cement with high contents of FA (40%) or SG (60%). Table 1 provides the chemical composition of the cement and SCMs along with their physical characteristics. Well-graded river sand with fineness modulus, specific gravity, and absorption of 2.9, 2.53, and 1.5%, respectively, was used as fine aggregate. Natural siliceous gravel (nominal size of 9.5 mm) was used as coarse aggregate. Absorption and specific gravity of gravel were 2% and 2.65, respectively. For slab specimens subjected to salt–frost scaling exposure, an air-entraining admixture was added during mixing to achieve a target fresh air content (6 ± 1%). Preparation of specimens followed ASTM C192/C192M [28]; standard curing (20 ± 2 °C and relative humidity [RH] > 95%) was started 24 h after casting and continued for 56 days to allow for the pozzolanic reactivity of FA and SG, as directed by CSA A23.1/A23.2 [29]. Details on mixture proportions and associated compressive strength as per ASTM C39/C39M [30] are given in Table 2.

#### 2.1.2. Surface Coatings

Neat ethyl silicate (ES) and high-molecular-weight methacrylate (HM) were used as dispersion media for nanoparticles. These polymer-based resins were chosen based on results obtained previously by the authors [12]. ES could resist aggravated PSA exposure when applied in two stages separated by 1 h, and each cycle involved three successive applications at 15 min time intervals. In contrast, HM failed to protect concrete under the same environment. These findings stimulated the current study, where nanoparticles were incorporated in an attempt to decrease the number of application layers and/or enhance the efficiency of these commonly used resins as measures for protection of concrete against severe conditions. Neat coatings were applied in accordance with manufacturer recommendations to provide a comparative reference to nanocomposites. Hence, ES was coated using a brush in one cycle and only composed of three layers separated after 15 min. HM resin was initially mixed with a promotor, followed by adding an initiator, stirring for 2 min, and then brushing in two layers separated after 16 h. 

Montmorillonite- and halloysite-based nano-clays (denoted as MC and HC, respectively) were incorporated alternatively into base resins to synthesize four nanocomposites: ES/MC, ES/HC, HM/MC, and HM/HC. Montmorillonite nano-clay particles had 99% purity and ranged in size between 40–80 nm, with an average specific surface area and density of 70 m^2^/g and 1.98 g/cm^3^, respectively; particles were organically modified using MT2EtOH (methyl, tallow, bis-2-hydroxyethyl, quaternary ammonium) organic modifier at a concentration of 90 meq/100 g. Comparatively, halloysite nano-clay (90% purity, density of 2.53 g/cm^3^ and average specific surface area of 64.2 m^2^/g) possessed a tube structure with average length, inner diameters, and outer diameters of 2 µm, 20 nm, and 200 nm, respectively. Two loading ratios of MC and HC were incorporated (2.5% and 5% by mass) in each nanocomposite. Synthesis of composites started with adding the powder to the base resin and stirring using a mixing drill/homogenizer (1000 rpm) until reaching uniform dispersion of powder without any visible agglomerations. Subsequently, ultrasonication was performed at lab ambient conditions for an hour in an ultrasonic bath at 200 W and 60 kHz to achieve homogenous dispersion quality of the nanoparticles in each composite [31]. 

Cured specimens were allowed to air dry for 48 h and then treated with each nanocomposite following the same procedure as neat resins. Subsequently, coated specimens with nanocomposites were cured for additional 7 days at standard conditions (20 ± 2 °C and RH > 95%) to permit pozzolanic reactivity of nanoparticles with substrates, if any. After curing, coated specimens were wire-brushed after drying to remove any excess coating on the surface of samples to simulate surface wearing of concrete by external sources (e.g., mechanical attrition).

### 2.2. Experiments

#### 2.2.1. Characterization of Coatings and Concrete Surface

Transmission electron microscopy (TEM) was conducted on ultrathin film samples prepared from the previously ultrasonicated nanocomposites to verify the dispersion quality. The potential enhancement in the penetrability of concrete due to coating with nanocomposites was evaluated using rapid chloride penetration testing (RCPT) according to ASTM C1202 [32] relative to untreated specimens and concrete coated with neat resins. Duplicate specimens from each mixture were tested with only the side facing the cathodic part coated to specifically determine the penetration resistance through this surface. Specimens were axially split right after testing and sprayed using 0.1 M silver nitrate solution, which converts to whitish silver chloride precipitate and provides a measurable penetration depth of chloride ions into the surface; this depth was found to reliably correlate with the physical nature of pore structure [33]. 

Moreover, the influence of nanocomposites on the rates of salt solution supply and evaporation through the evaporative front, which controls the deterioration level of concrete subjected to wetting/drying exposures (e.g., PSA), was determined through a designed absorption/desorption test. The experiment was performed on duplicate cylindrical disks (75 × 50 mm) from each mixture/coating using the same salt brine (10% Na_2_SO_4_) as implemented in the PSA exposure conditions. All sides of specimens were sealed with a rapid-setting epoxy followed by coating of the top surface with the corresponding neat resin/nanocomposite following the procedure described earlier. For absorption, samples were oven-dried at 50 ± 2 °C and 40 ± 5% RH until a constant mass was achieved (<0.1% mass variation in two consecutive days), while specimens for desorption were fully submerged in 10% Na_2_SO_4_ until constant mass, noting that drying and saturation primarily occurred through the uncoated bottom face. Subsequently, the bottom surface of specimens was coated with two epoxy layers to avoid further drying, and the initial mass (*m*_0_) was determined. This procedure would then allow for movement of salt brine through the coated surface for further drying saturation or drying of specimens in the absorption and desorption experiments, respectively. Next, in case of absorption, dry specimens were fully immersed in 10% Na_2_SO_4_ for 48 h, while for desorption, specimens were dried at 40 ± 2 °C and 35 ± 5% RH (same drying condition as PSA exposure) for 48 h. The percentages of absorption and desorption were determined as follows:

Absorption or Desorption (%) = [(*m*_48_ − *m*_0_)/*m*_0_] × 100 (1)

where *m*_48_ is the mass (g) of specimens at 48 h and *m*_0_ is the initial mass of specimens (g), including the epoxy sealer. 

#### 2.2.2. Harsh Exposures

Triplicate specimens from uncoated and coated concrete were subjected to accelerated PSA testing conditions introduced by Bassuoni and Rahman [34], which presented conclusive trends on the vulnerability of concrete to PSA in 120 days. One-third of each specimen (75 × 150 mm) was partially immersed in a 50 mm plastic container filled with 10% Na_2_SO_4_ solution. The upper part of the cylinders was exposed to cyclic temperature and RH conditions; each cycle continued for 24 h with 8 h of dry phase (40 ± 2 °C and 35 ± 5% RH) followed by 16 h of humid phase (20 ± 2 °C and 90 ± 5% RH). These conditions promote cyclic conversion between the dry phase of sodium sulfate [thenardite (Na_2_SO_4_)] and its hydrate phase [mirabilite (Na_2_SO_4_·10H_2_O)], increasing the supersaturation ratio of solution. The salt solution was replenished during exposure with fresh brines every 30 cycles. The final condition of specimens was given a visual rating based on a pictorial rating scale (from 0 to 5 [no to maximum scaling]). In addition, the mass change of specimens relative to the initial mass was calculated every 30 cycles.

The resistance of coated concrete specimens (250 × 250 × 100 mm) was also evaluated against salt–frost scaling as per ASTM C672/C672M [35] using 4% CaCl_2_ salt solution ponded on the top surface of specimens. The test involved 50 cycles, where each cycle consisted of two stages: 16 h freezing stage (−18 °C) followed by 8 h thawing phase (22 ± 2 °C and 50 ± 5% RH). Scaled concrete was collected, and its cumulative mass was taken as a measure of the damage level. In addition, specimens were visually assessed according to ASTM C672/C672M [35].

#### 2.2.3. Thermal and Microstructural Analysis

The alteration of concrete components due to surface treatment and deterioration from exposures was investigated using thermal and microstructural techniques. Differential scanning calorimetry (DSC) was applied to quantify mineral phases of powder samples prepared from extracted chunks from the outer layer (0 to 5 mm) of unexposed and exposed specimens. The powder was prepared by pulverizing fractured pieces to a fine powder passing through sieve #200 (75 μm). In addition, microanalysis by scanning electron microscopy (SEM) equipped with energy-dispersive X-ray analysis (EDX) was performed to support DSC results. For this purpose, chunks were extracted from the reaction front and carbon coated to improve sample imaging. In the EDX spectra of SEM results, the identified elements (green color) are represented by their designation in the periodic table (e.g., Ca: calcium, Si: silicon, Na: sodium, etc.). Each element family consists of two recognizable lines ka and kb (red color) appended to the chemical symbols. The ratio of intensities of the ka and kb peaks is approximately 10:1; when the peaks are resolved by EDX, this ratio should be apparent in the identification of an element, which is translated to the identified green peak. 

## 3. Results

### 3.1. Dispersion of Nanocomposites

**Figure 1:**
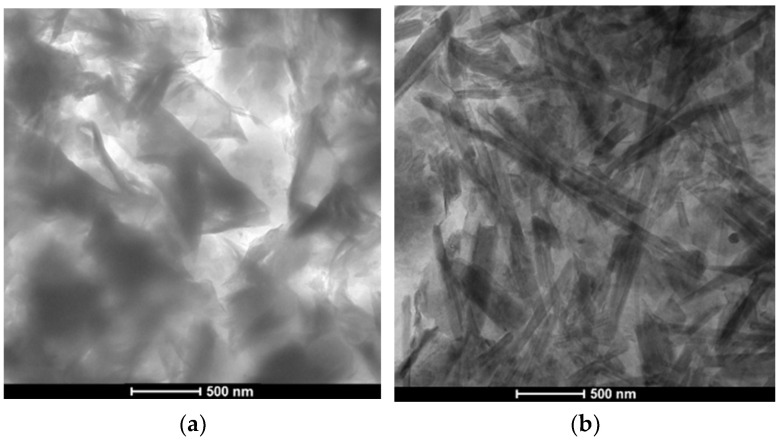

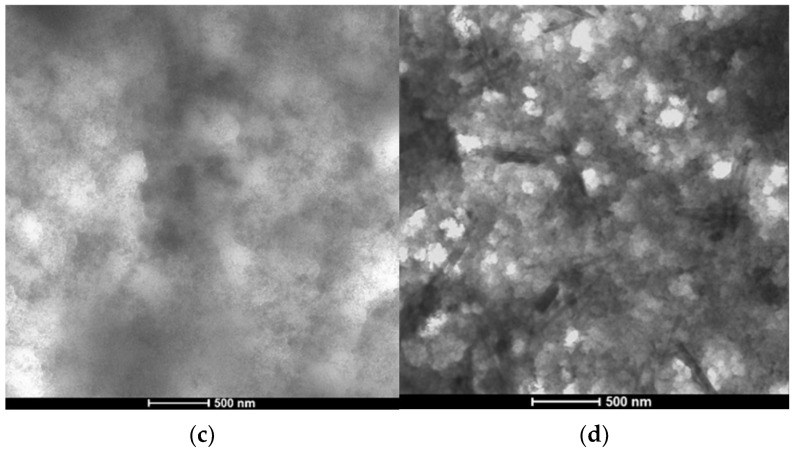
Exemplar TEM images of: (**a**) ES/MC, (**b**) ES/HC, (**c**) HM/MC, and (**d**) HM/HC composites at 2.5% concentration.

A TEM analysis was applied to evaluate the morphology of developed composites at the nanoscale due to nanoparticle inclusion. Figure 1 presents exemplar TEM visualizations for all composites prepared at a loading ratio of 2.5%. Particles of montmorillonite nano-clay showed homogeneous distribution in ES and HM neat resins, without significant agglomerates, forming as exfoliated sheets with the development of some intercalated stacks of nano-clay in case of ES. HM/MC, however, showed a relatively more coherent matrix since resin showed a higher integration with nanoparticles. In both cases, the interwoven-like morphology might boost the penetration resistance of ES/MC and HM/MC nanocomposites. Comparatively, halloysite nano-clay appeared as crossing hollow tubes varying in length but uniformly dispersed in the neat resins. Few agglomerates were formed at some locations within ES that did not compromise the performance of the developed nanocomposite. Similar to MC, halloysite nanoparticles were accommodated by HM resin, appearing as a fully integrated system.

### 3.2. Mass Transport Properties

#### 3.2.1. RCPT

Table 3 summarizes the obtained results of RCPT (average passing charges and corresponding penetration depths) for uncoated and coated specimens. Testing of uncoated specimens terminated prior to the scheduled period of the test (6 h) due to overheating caused by the Joule effect. Thus, migration coefficients were calculated according to NT BUILD 492 [36] to provide a uniform comparison. Migration coefficients and penetration depths showed a direct correlation to passing charges. Reference (uncoated) specimens had the least resistance to chloride ingress in terms of passing charges, penetration depths, and migration coefficients. The penetration depth was 50 mm (entire sample depth) for all uncoated disks regardless of the mixture design, yet the FA and SG samples did not experience overflow as a GU mixture due to the biased impact of the high replacement ratio of SCMs on reducing the electrical conductivity of concrete [33], resulting in 33% less chloride migration coefficients [47.8 × 10^−12^ m^2^/s] for FA and SG mixtures compared to GU [71.7 × 10^−12^ m^2^/s].

The application of neat coatings moderately enhanced the penetration resistance of specimens compared to reference concrete. For example, ES reduced penetration depths of GU, FA, and SG specimens by 61%, 54%, and 43%, respectively. Compared to ES, specimens coated with HM showed moderately higher chloride ingress; for instance, the penetration depth of the GU specimens coated with HM was 24.5 mm relative to 19.5 mm for the same specimens coated with ES (i.e., 25% increase). Blending nanoparticles with neat coatings (MC or HC) further reduced the transport properties of concrete regardless of the mixture components. For instance, the passing charges and migration coefficient of GU specimens coated with ES/MC2.5 were 935 coulombs and 6.5 × 10^−12^ m^2^/s, respectively, compared to 2773 coulombs and 18 × 10^−12^ m^2^/s for ES-coated specimens (66% and 63% reduction, respectively). An analysis of variance (ANOVA) at a significance level (*α*) of 0.05 [37] (Table 4) confirmed the statistical significance effect of using ES/MC2.5 relative to neat ES on migration coefficients where an *F*-value of 330.8 was obtained compared to a critical threshold (*Fcr*) of 18.51. The use of halloysite nano-clay with ES led to further reduced transport properties compared to MC at both loading ratios. For example, coating GU specimens with ES/HC2.5 provided 16% and 21% less passing charges and migration coefficients compared to conjugate specimens coated with ES/MC2.5; however, the ANOVA revealed no statistical significance for changing the type of nanoparticles with ES at both dosages [*F*-value of 11.46 compared to *Fcr* of 18.51 (Table 4)]. Increasing the dosage of nanoparticles (MC or HC) resulted in less efficiency of the nanocomposites, where GU specimens coated with ES/MC5 and EC/HC5 had 37% and 19% higher migration coefficients compared to ES/MC2.5 and EC/HC2.5, respectively, with statistical significance.

Similar to ES-based nanocomposites, mixing nanoparticles with HM significantly improved the resistance of concrete to fluid ingress relative to plain HM, with halloysite nano-clay performing better than montmorillonite nano-clay. For example, the passing charges of GU specimens coated with HM/MC2.5 and HM/HC2.5 were 840 and 797 coulombs (78% and 80% reductions compared to neat HM) and had corresponding migration coefficients of 6.5 × 10^−12^ and 5.6 × 10^−12^ m^2^/s, respectively (71% and 75% reductions compared to neat HM). Nevertheless, the ANOVA showed that changing the type of nanoparticles had an insignificant effect on the migration coefficient; for example, at a 2.5% dosage, an *F*-value of 13.23 was obtained compared to an *Fcr* of 18.51 (Table 4). For both types of nanoparticles, increasing the concentration within the matrix significantly reduced the penetration resistance of concrete, which was also confirmed by an ANOVA (Table 4). For instance, specimens coated with HM/HC5 experienced almost double the passing charges of specimens coated with HM/HC2.5. 

The use of HM-based nanocomposites provided comparable results to ES-based nanocomposites for both types of nanoparticles at the lower dosages of 2.5%. For example, GU specimens coated with ES/MC2.5 and ES/HC2.5 had passing charges of 935 and 786 coulombs, respectively, compared to 840 and 797 coulombs for HM/MC2.5 and HM/HC2.5. At a higher dosage of nanoparticles (5%), ES-based nanocomposites showed less transport properties than HM-based nanocomposites regardless of the types of nano-clay.

#### 3.2.2. Absorption/Desorption

**Figure 2:**
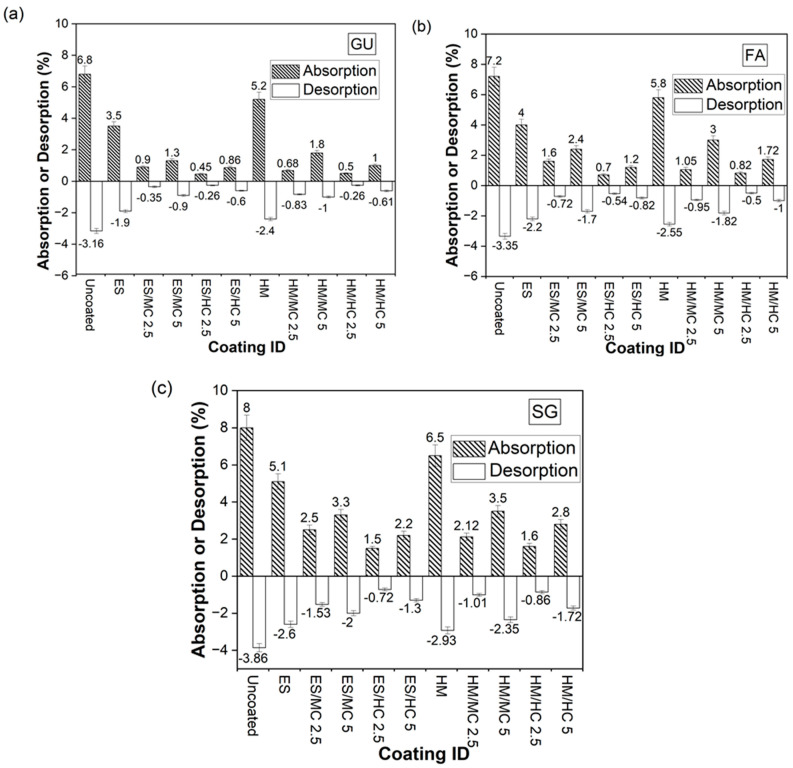
Absorption and desorption percentages of concrete specimens from the (**a**) GU, (**b**) FA, (**c**) SG groups. (Note: All data are listed in Appendix Table A1).

Figure 2a–c (detailed data are presented in the Appendix Table A1) shows the results of absorption/desorption for uncoated and coated samples to indicate the effect of surface treatment on the readiness of fluid transport through the pore structure of concrete, which controls the rate of concrete disintegration under PSA. Conforming to RCPT trends, reference concrete experienced the maximum absorption and desorption ratios, with SCMs increasing the fluid transport through wicking. Absorption percentages of uncoated GU, FA, and SG specimens were +6.8%, +7.2%, and +8.0%, respectively, and their corresponding desorption ratios were −3.2%, −3.4%, and −3.9%, respectively. Neat ES provided moderate improvements in solution uptake and evaporation behaviors for all mixtures compared to lower levels of enhancement for HM. For instance, GU specimens coated with ES had 48% and 34% less absorption and desorption ratios, respectively, compared to 23% and 24% for HM-coated specimens. 

For ES/nanocomposites, the best performance occurred for composites containing 2.5% HC, and this was replicated for all concrete mixtures. The application of ES/HC2.5 on GU concrete, for example, significantly reduced the absorption and desorption percentages by 87% and 75%, respectively, compared to ES. This effect was deemed significant by the ANOVA (Table 4), where an *F*-value of 464 was obtained compared to an *F_cr_* of 18.51. Using MC at the same dosage improved the resistance to absorption and desorption compared to neat ES but yielded higher penetrability than HC; for example, double the absorption of GU specimens coated with ES/MC2.5 was obtained relative to the conjugate specimens coated with ES/HC2.5. Thus, the ANOVA depicted that alternating the type of nanoparticles within ES had a significant effect at a dosage of 2.5%. Increasing the amount of both nanoparticles (5%) in neat ES showed an inferior performance compared to nanocomposites prepared using lower dosages (2.5%), with statistical significance (Table 4).

Similar to ES, the lowest penetrability for HM/nanocomposites was achieved using halloysite nano-clay at a dosage of 2.5% for all specimens. HM/HC2.5 yielded absorption and desorption ratios of +0.50% and −0.26%, respectively, for GU concrete, which represented a 90% reduction in transport properties compared to specimens treated with neat HM. An ANOVA confirmed the remarkable effect of using 2.5% HC, where *F*-values of 206 and 864 were calculated for absorption and desorption, respectively. Replacing halloysite nano-clay with montmorillonite nano-clay at the same dosage (2.5%) led to the same findings as in the case of ES, where a higher solution ingress/evaporation was obtained without statistical significance for absorption and with significance for desorption. Employing higher amounts of nanoparticles (5%) into HM decreased the functionality of nanocomposites in terms of absorption and desorption percentages regardless of their type. For example, using HM/HC5 with GU specimens instead of HM/HC2.5 doubled the absorption ratio and increased the desorption ratio by 134%; the ANOVA verified the significance of such a replacement (Table 4). 

### 3.3. Durability Exposures

#### 3.3.1. Physical Salt Attack (PSA)

**Figure 3:**
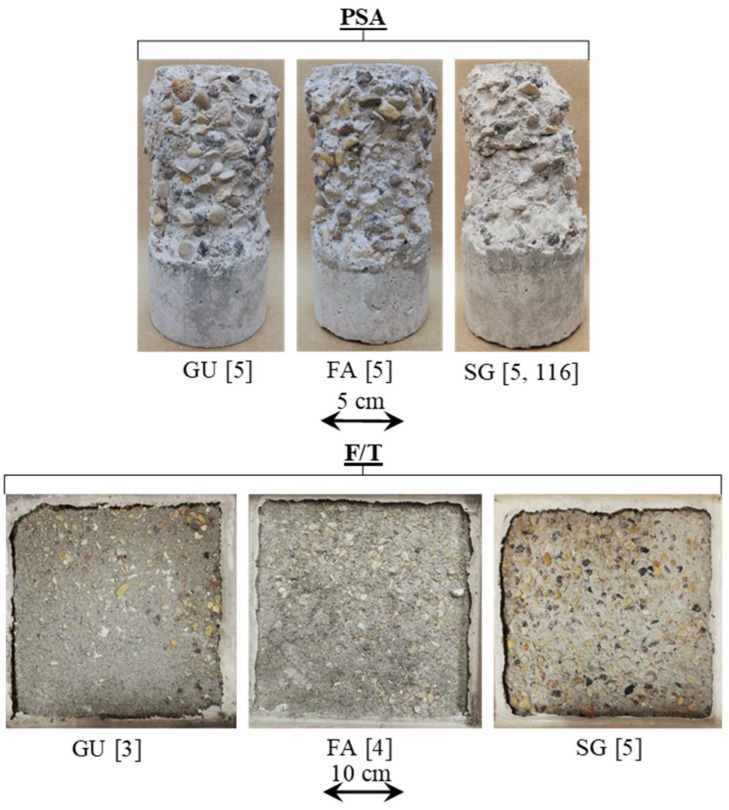
Reference/uncoated specimens at the end of the PSA and salt–frost scaling exposures (Note: Numbers between brackets are the final visual ratings).

**Figure 4:**
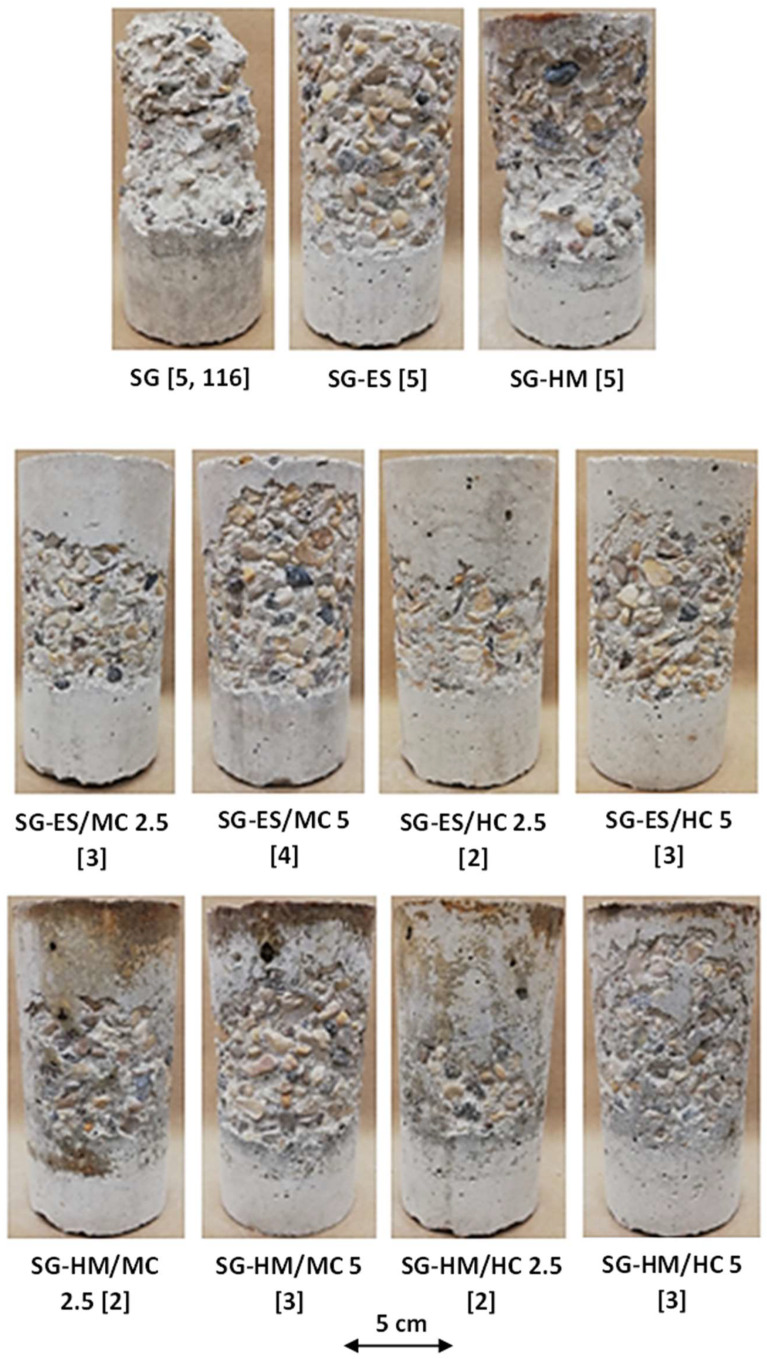
Uncoated and coated SG specimens after 120 days/cycles of PSA exposure (Note: Numbers between brackets are the final visual ratings; SG specimens failed at 116 days).

**Figure 5:**
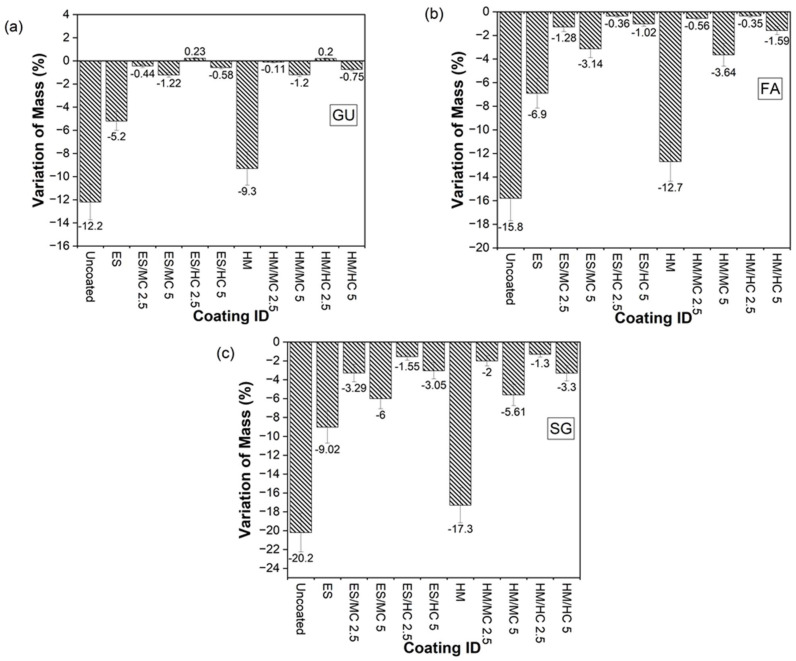
Cumulative mass variation after the PSA exposure from the (**a**) GU, (**b**) FA, (**c**) SG groups (Note: All data are listed in Appendix Table A2).

Figure 3 displays the visual condition of untreated specimens after the PSA exposure, and Figure 4 shows the various visual state of untreated and treated SG specimens after exposure since they suffered the most damage; specimens were evaluated visually according to a pictorial visual rating scale proposed by [34]. In addition, the cumulative mass variation (gain or loss) of specimens after the PSA exposure, and detailed data measured every 30 cycles of exposure are given in Figure 5a–c and Appendix (Table A2), respectively. All damaged specimens had an intact submerged portion in salt solution and lost mass only from upper portions, which is a typical feature of PSA [38]. Deterioration proceeded progressively where salt depositions accumulated on and below the surface layer in the drying part (above solution) of specimens, followed by scaling and peeling that layer to an extent determined by the type of base mixture and coating. Reference/uncoated specimens of the three mixtures yielded the greatest damage, with all specimens having a visual rating of 5 (max. level); using high contents of SCMs (40% FA or 60% SG) increased the vulnerability of specimens for damage. The final mass loss for GU, FA, and SG specimens was 12.2%, 15.8%, and 20.2%, respectively, with SG specimens fractured after 116 cycles. 

The application of neat coatings assisted with discounting the progression and level of damage; ES served better than HM regarding this purpose, as shown in Figure 4 for SG specimens. Using ES decreased the mass loss of such specimens from 20.2% to 9.0% (i.e., 55% enhancement) compared to 17.3% for HM (14% enhancement). Nevertheless, both neat coatings proved unsatisfactory for resisting PSA conditions imposed by testing, as depicted by the visual condition and mass loss. Using coatings modified with nanoparticles drastically altered the performance of concrete. Using ES/nanocomposites delayed the onset of surface scaling, keeping all specimens intact for at least half the testing period, and, accordingly, reduced the final mass loss remarkably. For instance, SG specimens coated with ES/MC2.5 had a final mass loss of 3.3%, which is 63% less than the damage occurred in specimens coated with ES. This significant improvement was confirmed statistically since an *F*-value of 101.38 was obtained compared to an *F_cr_* of 7.71 (Table 4). Nanocomposites with halloysite nano-clay outperformed their counterparts produced with montmorillonite-based clay for all cases. SG specimens, for example, coated with ES/HC2.5 had approximately 50% less mass loss compared to ES/MC2.5. Increasing the dosage of both MC and HC in neat ES reduced the efficiency of the nanocomposites. For example, GU, FA, and SG cylinders protected with ES/MC5 lost masses of 1.2%, 3.1%, and 6.0%, respectively, compared to 0.6%, 1.0%, and 3.1% for same specimens treated with ES/MC2.5. A comparable trend was observed for specimens treated with ES/HC composites.

Similarly, specimens coated with HM nanocomposites could protect concrete from aggressive PSA conditions with similar correlations as ES nanocomposites. The lowest mass loss of 1.3% was achieved for specimens coated with HM/HC2.5, which indicates a remarkable enhancement of 93% in the resistance of SG specimens to PSA compared to the neat HM [*F*-value of 125.14 compared to *F_cr_* of 7.71 (Table 4)]. Respective specimens coated with HM/MC2.5 experienced a slightly higher mass loss (2.0%) without statistical significance. For both nanocomposite categories, incorporating a higher percentage of nanoparticles (5%) reduced the improvement in performance; for example, total mass reductions of 1.2%, 3.6%, and 5.6% were obtained for GU, FA, and SG specimens coated with HM/MC5, respectively, compared to 0.1%, 0.6%, and 2.0% for the same specimens treated with HM/MC2.5.

#### 3.3.2. Salt–Frost Scaling (F/T)

**Figure 6:**
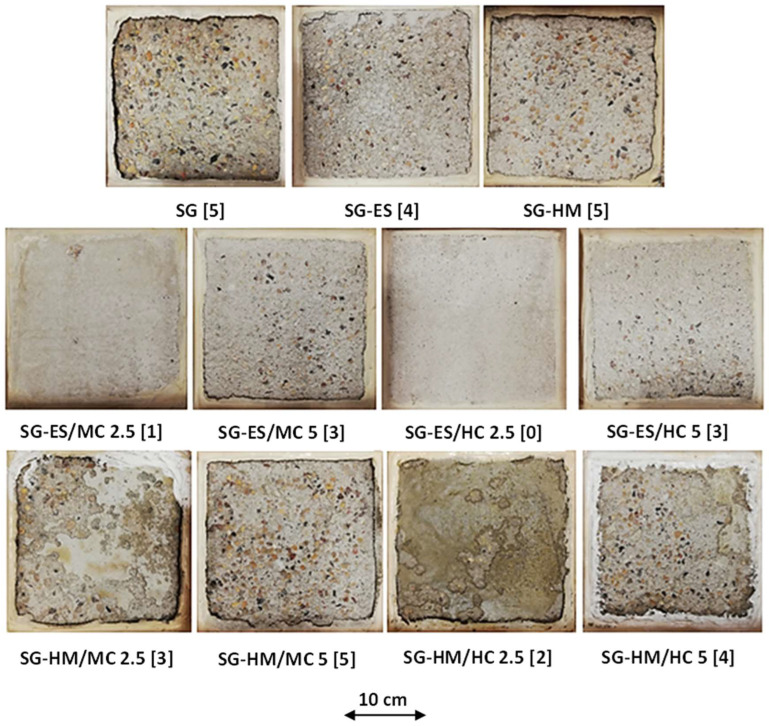
Uncoated and coated SG specimens at the end of the salt–frost scaling exposure (Note: numbers between brackets are the final visual ratings).

**Figure 7:**
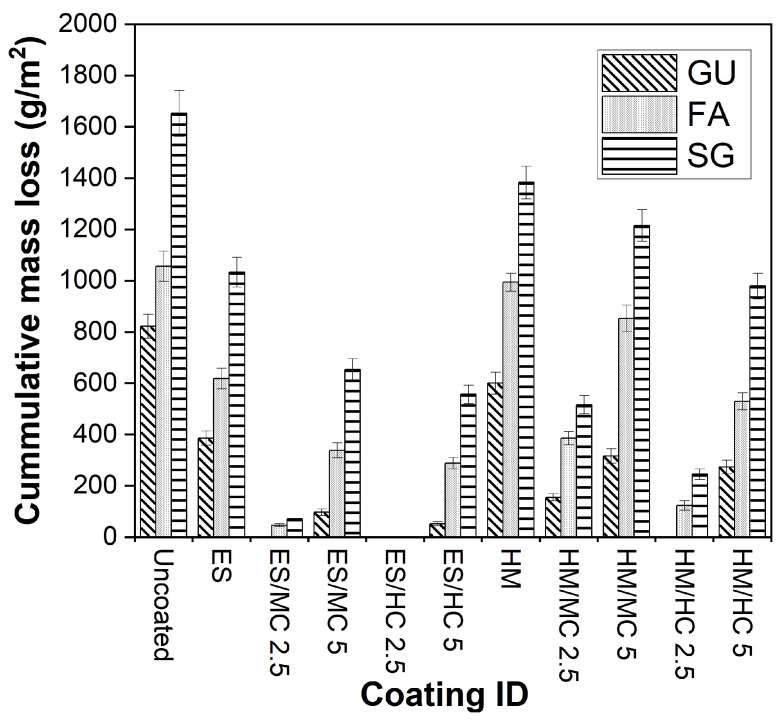
Cumulative mass loss of specimens at the end of the salt–frost scaling exposure.

Figure 3 and Figure 6 present examples of uncoated and coated SG slab specimens following a 50-day exposure involving freezing/thawing cycles combined with de-icing salts. Visual assessments according to ASTM C672/C672M (2012) are provided, and accumulated mass losses per square meter after 50 cycles are shown in Figure 7 (detailed data are listed in the Appendix Table A3). Uncoated slabs exhibited significant scaling and accelerated mass loss within the initial 10 cycles, resulting in final mass losses of 822, 1056, and 1652 g/m^2^ for GU, FA, and SG, respectively. These values exceed acceptance criteria set by many jurisdictions in Canada, such as the Ministry of Transportation of Ontario (MTO) [39] and Bureau du normalization du Quebec (BNQ) [40] failure limits for the salt–frost scaling test, which are 800 and 500 g/m^2^, respectively.

Applying neat coatings to exposed surfaces of slabs maintained the specimen integrity for a certain duration depending on the coating type and mixture constituents. However, following the detachment of the initial crust layer, all mixtures exhibited comparable performance to uncoated specimens. For instance, using ES moderately reduced surface scaling by 53% for GU but provided lesser improvements for FA and SG (average mass loss reduction of 39%). In contrast, HM exhibited inferior performance since the coating layer quickly detached before mid-exposure, resulting in final mass losses close to reference specimens (600, 994, and 1383 g/m^2^ for GU, FA, and SG, respectively), projecting inadequate protection in the field. 

Incorporating nanoparticles into neat coatings significantly enhanced concrete resistance against this exposure. For instance, compared to pure ES, all slabs coated with ES/HC2.5 remained completely intact (visual rating of 0) throughout the exposure with zero mass loss irrespective of mixture type. Similarly, replacing HC with MC yielded the same outcome for GU, while minimal mass losses (less than 71 g/m^2^) were observed for FA and SG, indicating remarkable improvements. Similar to the trends observed under the PSA exposure, increasing the nanoparticle dosage to 5% reduced the efficacy of nanocomposite functionality, particularly with FA and SG, demonstrating statistical significance when compared to samples coated with nanocomposites at a 2.5% loading ratio (Table 4). For example, the final mass losses of slabs coated with ES/MC5 were 97, 338, and 654 g/m^2^ for GU, FA, and SG, respectively. 

Regarding HM/nanocomposites, GU treated with HM/HC2.5 was the sole matrix that remained intact (visual rating of 0 and no mass loss) until the end of exposure. Their counterparts of FA and SG exhibited mass losses of 123 and 245 g/m^2^, respectively. In contrast to ES, changing the nanoparticle type with HM (i.e., MC instead of HC) led to substantial spalling (roughly three times) and demonstrated statistical significance, as indicated by the ANOVA (Table 4). For GU specimens, the application of HM/MC2.5 relative to HM/HC2.5 resulted in an *F*-value of 261.4 compared to a critical threshold (*F_cr_*) of 18.51. Increasing nanoparticles to 5% significantly impacted the effectiveness of nanocomposites against these harsh conditions for both nanoparticle types. Within the HM/nanocomposites group, samples treated with HM/MC5 showed maximum final mass losses of 316, 853, and 1215 g/m^2^ for GU, FA, and SG, respectively, indicating inadequate protection for the FA and SG concrete.

## 4. Discussion

### 4.1. Effect of Supplementary Cementitious Materials (SCMs)

As evident from the results, the reference (uncoated) specimens showed inferior performance in terms of pre-exposure characterizations (e.g., transport properties) and post-exposure performance, including visual assessment and mass loss. This underscores the susceptibility of unprotected concretes to severe conditions, as depicted in Figure 3. Nevertheless, the three mixtures displayed varying levels of deterioration; the utilization of the high replacement ratios of SCMs, specifically 40% FA and 60% SG, exacerbated the susceptibility of concrete to both PSA and salt–frost scaling exposures, with FA demonstrating a relative advantage over SG. The findings of this study align with prior research conducted under similar test conditions. For instance, Sakr and Bassuoni [41] found that a higher cement replacement ratio with SCMs increased absorption, total porosity, and wicking factors along with decreased micropores (<0.1 μm) and compressive strengths. These factors led to slower microstructural and mechanical property evolution over time compared to GU specimens with similar binder content and *w*/*b* and thus higher vulnerability to surface scaling.

**Figure 8:**
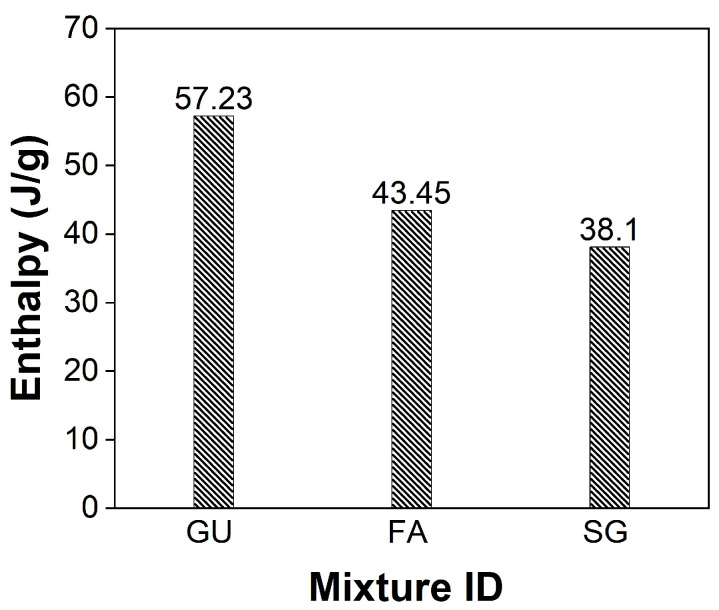
Enthalpy of initial portlandite in unexposed reference samples at 56 days (Note: accuracy of measurement is ±2%).

**Figure 9:**
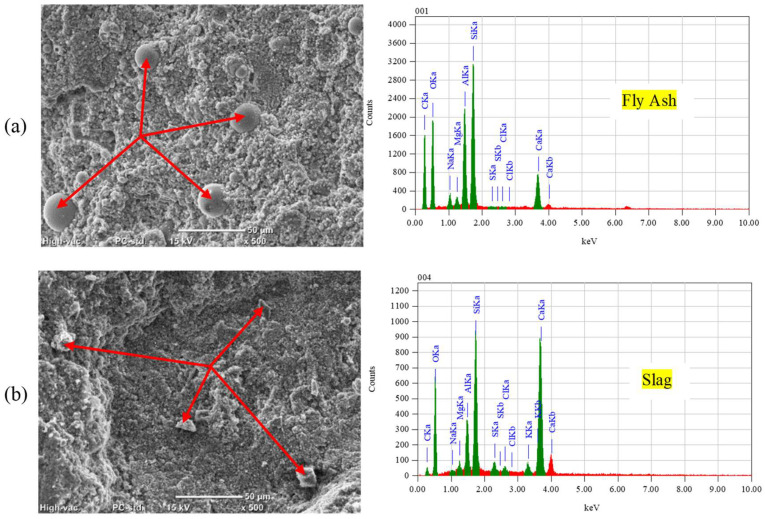
SEM and EDX analyses for unexposed specimens: (**a**) FA and (**b**) SG.

To investigate changes in hydration products, a DSC analysis was conducted on the outer layer of unexposed specimens after the 56-day moist curing period. The enthalpy concept, specifically the heat flow integration of peaks over the temperature range spanning from 400 and 450 °C, was employed to quantitively assess portlandite content (Figure 8). DSC results suggested in the cases of fly ash and slag notable reductions of 24% and 34% in portlandite content, respectively, compared to GU specimens. These reductions may be attributed to the pozzolanic reaction and high content of SCMs in the binary blended binders, indicating a lower degree of maturity at 56 days compared to GU cement [42,43]. An SEM analysis corroborated these DSC findings, revealing the presence of significant amounts of unbonded particles that not only compromised transport properties and microstructure of concrete but also easily detached during exposures (Figure 9).

**Figure 10:**
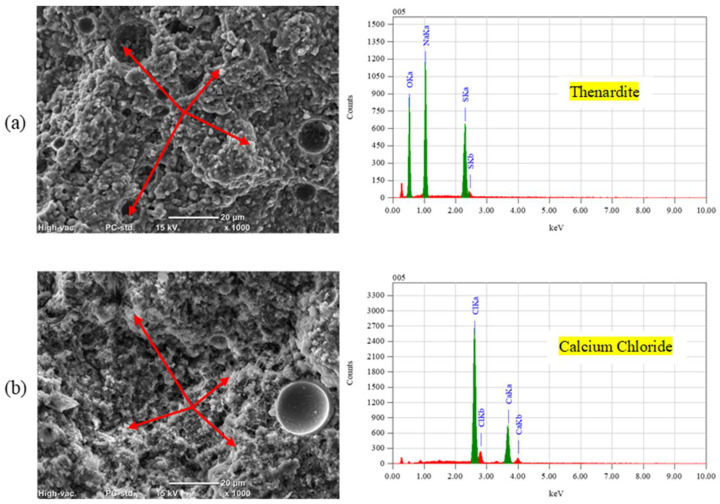
SEM and EDX analyses for exposed FA specimens: (**a**) PSA and (**b**) F/T.

In the case of PSA, both fly ash and slag exhibited increased mass losses of 30% and 66%, respectively, compared to GU specimens, with slag samples fracturing before the exposure concluded. During salt–frost scaling exposure, due to the ease of salt solution ingress and the readiness of unbonded particles to detach, FA and SG showed higher surface scaling and total mass losses (increases of 28% and 100%, respectively) compared to GU. Figure 10 illustrates salt crystals filling voids previously occupied by loose fly ash particles, which easily dislodged. Moreover, within the deteriorating zone (0–10 mm) from the surface, significant amounts of thenardite (sodium sulfate) in PSA and calcium chloride in salt–frost scaling crust layers were detected, which were primarily responsible for the progression of damage. 

### 4.2. ES-Based Nanocomposites

The utilization of a single cycle (comprising three successive saturating applications) of neat ethyl silicate resulted in certain improvements, including better visual assessments and reduced mass losses, in both exposure conditions compared to the untreated samples. Sakr et al. [12] documented significant enhancements with neat ethyl silicate through six successive saturating applications for concrete made with GU cement, attributing these improvements to the dual functions of ethyl silicate (pore blocking and water repelling). Hence, the comparatively lower performance observed in this study was attributed to the use of only three layers (evaluating ethyl silicate functionality with half of the treatment quantity) and the examination of mixtures prepared with binary blended binders containing SCMs, confirming the infeasibility of this reduction in the number of layers if ES is used without nano-modification. 

**Figure 11:**
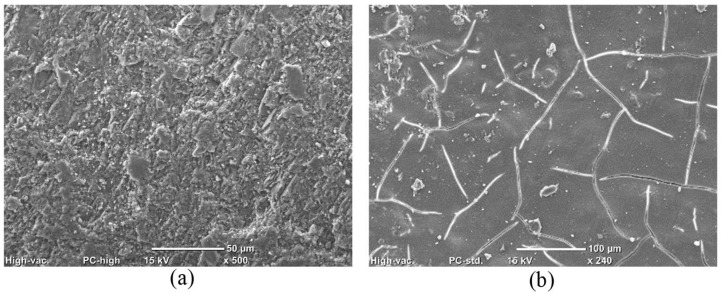
SEM images for surfaces of unexposed GU specimens coated with (**a**) ES and (**b**) HM.

**Figure 12:**
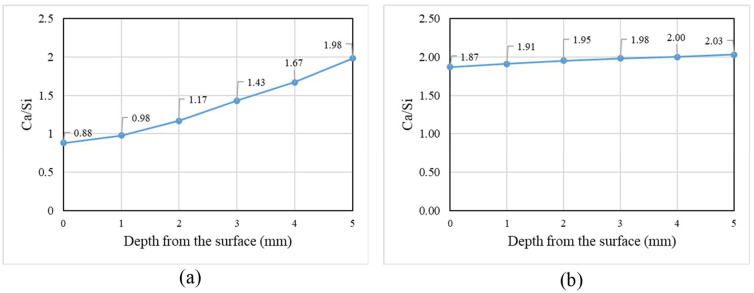
*Ca*/*Si* curves representing the first 5 mm of GU samples coated with (**a**) ES and (**b**) HM.

A nano-indentation test was employed to investigate the protective mechanisms of coatings. Five rows spaced at 1 mm intervals starting from the outer surface of the coated sample were examined. Within each row, five points (spaced at 50 μm) underwent indentation, and the results were averaged to represent the hardness value. The surface hardness of GU samples coated with ES were increased, particularly within the first 3 mm from the surface (from 0.85 to 0.97 GPa), indicating effective penetration and reaction of silicates with portlandite forming C-S-H, leading to a denser microstructure (Figure 11a). SEM equipped with EDX confirmed the nano-indentation results, revealing a gradual decrease in Ca/Si within the reaction front (0–5 mm) moving inwards from 0.88 to 1.98 (compared to 2.05 in the sample core), as shown in Figure 12a. ES demonstrated greater success with GU than the other two mixtures due to the higher initial content of calcium hydroxide in the cement paste after 56 days (Figure 8), facilitating the formation of calcium silicate hydrate and resulting in a denser microstructure. Additionally, the average absorption and desorption percentages moderately decreased by 43% and 35%, respectively. However, these improvements were not sufficient, as the samples were not fully protected and exhibited significant disintegrations. For example, ES-coated samples exposed to PSA conditions surpassed the moderate scaling level (3% [41]), with mass losses of 5.2%, 6.9%, and 9.0% for GU, FA, and SG, respectively. Similarly, in salt–frost scaling exposure, FA and SG failed to meet MTO and BNQ municipal standards, with total mass losses of 618 g/m^2^ and 1033 g/m^2^. 

**Figure 13:**
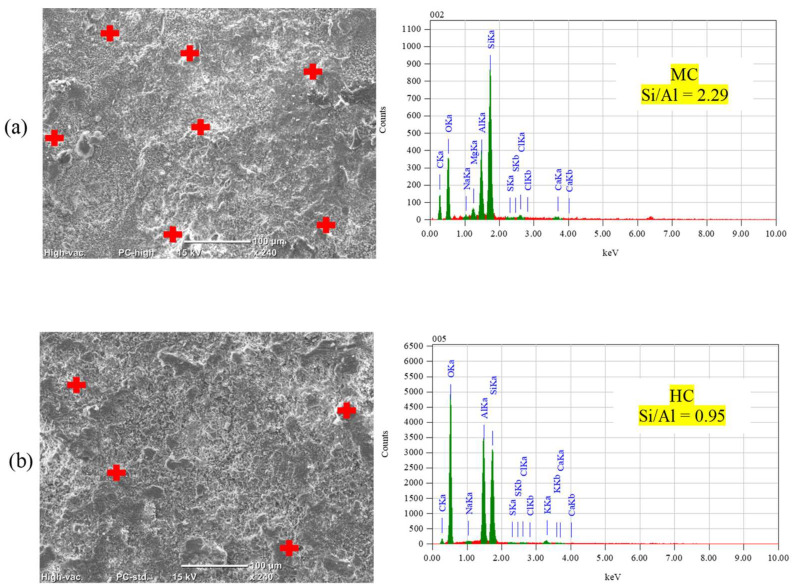
SEM and EDX analyses for surfaces of GU specimens coated with (**a**) ES/MC 2.5 and (**b**) ES/HC 2.5.

In the context of PSA, the limited effectiveness of ES was attributed to its relatively high absorption and desorption percentages (Figure 2), enabling a continuous salt solution supply, leading to progressive salt crystallization and increased peeling. For the salt–frost scaling exposure, ES did not fully prevent chloride ingress (chloride penetration depths of 19.5 mm, 23 mm, and 28.5 mm for GU, FA, and SG, respectively), causing cumulative calcium chloride deposition, salt crystallization, and progressive spalling in concrete. In contrast, all ES/nanocomposites outperformed neat ES, exhibiting lower mass losses and improved visual assessments in both exposures irrespective of the mixture design (Figure 4 and Figure 6). Enhanced functionalities, particularly in terms of transport properties, were achieved with the lower loading ratio of nano-clay (2.5%) for both MC and HC types due to reduced agglomeration and heterogeneous diffusion observed at higher nano-clay amounts per unit volume of resin. ES/HC nanocomposites demonstrated better improvements than MC nanocomposites at the same dosage, credited to the unique nanotube shape of HC particles, allowing for better interlocking with the resin compared to the solid platelets of MC (Figure 1). Additionally, the smaller particle size of MC (average of 40–80 nm) relative to HC (average of 200 nm) resulted in more lumps per unit volume of resin [44]. Figure 13 illustrates examples of surfaces of GU samples coated with ES/HC2.5 and ES/MC2.5, showcasing a more even surface in the case of halloysite nanocomposites at the same dosage. 

**Figure 14:**
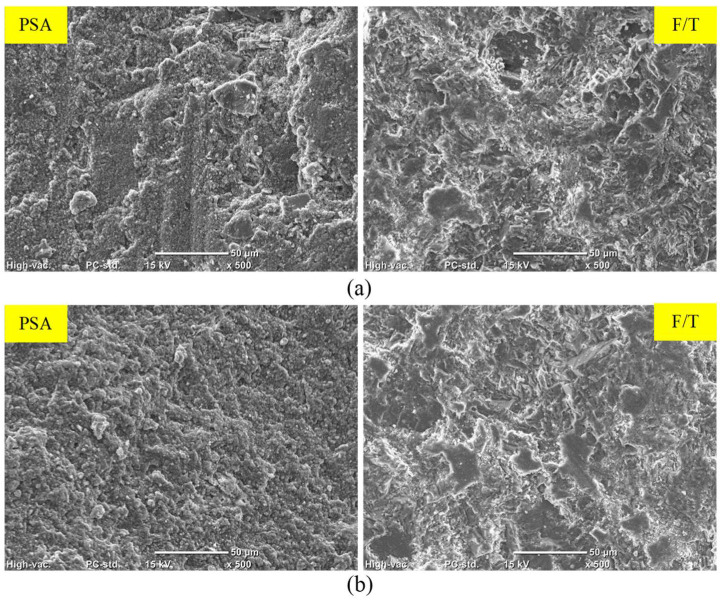
SEM images for SG specimens coated with (**a**) ES/MC 2.5 and (**b**) ES/HC 2.5 after both exposures.

The enhancements observed in ES/nanocomposites primarily stem from the inherent features of ES (pore blocking and water repelling) and the improved barrier properties achieved through nanoparticle incorporation. ES nanocomposites based on MC exhibited an interwoven structure (Figure 1a) on the concrete surface, significantly mitigating the transport of salt solution (both intake and evaporation) within concrete (Figure 2). This resulted in a reduction in the wicking factor of each matrix, a crucial indicator of the concrete resistance against PSA, and led to a notable decrease in chloride ingress into concrete (Table 3). The wicking factor reflects the connectivity and tortuosity of the pore structure, which determines the ease of the solution transport into or out of concrete by wicking [41]. Figure 14a illustrates limited or no presence of sodium sulfate and calcium chloride in SG (most vulnerable) samples after both exposures. Similarly, halloysite-based nanocomposites formed a fishnet shape (Figure 1b) on the concrete surface, potentially controlling the ease of the salt solution ingress into the material. This resulted in nearly intact concrete specimens (Figure 4 and Figure 6) with minimal amounts of sodium sulfate and no calcium chloride observed after PSA and salt–frost scaling, respectively, as depicted in Figure 14b.

### 4.3. HM-Based Nanocomposites

**Figure 15:**
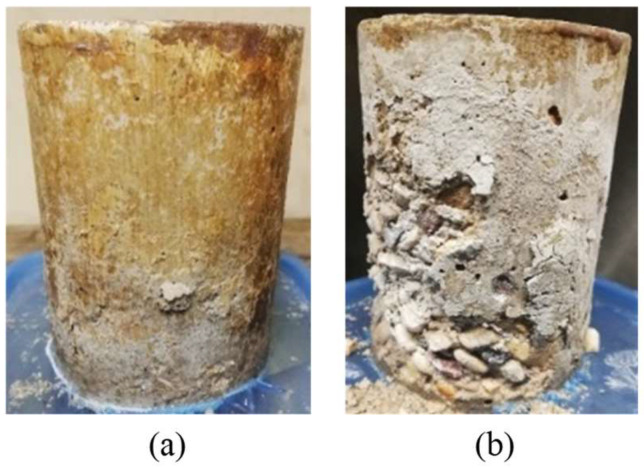
HM detachment from the GU specimens after (**a**) 15 and (**b**) 45 cycles.

HM is primarily utilized as a crack filler due to its capacity to partially fill superficial voids and cracks, forming a thin layer on the surface of concrete, isolating concrete from surrounding contaminants [45]. Nanoindentation results indicated minimal variations in surface hardness, eliminating the possibility of any significant reaction with the substrate. This was further corroborated by nearly unchanged Ca/Si within the first 5 mm of the reaction front, as depicted in Figure 12b. The efficiency of HM was insufficient at protecting concretes with high *w*/*b* from PSA due to diminished adhesion (bond strength) between the treatment and the substrate at higher moisture levels in concrete pores, which led to detachment after 15 cycles (Figure 15), resulting in merely 18% less mass loss compared to uncoated concrete. 

In the case of salt–frost scaling, HM layers detach before 25 cycles, causing surface scaling similar to uncoated surfaces. Slabs coated with neat HM exhibited high mass losses (600 g/m^2^, 994 g/m^2^, and 1383 g/m^2^ for GU, FA, and SG, respectively), surpassing the BNQ failure limit. The effectiveness of HM was compromised by shrinkage cracks on coated surfaces (Figure 11b), enabling the easy transport of surrounding contaminants through the material. For PSA, these cracks led to elevated absorption and desorption values; for example, GU samples coated with neat HM showed values (5.20% and 2.40%, respectively) close to uncoated specimens. The high liquid transport facilitated sodium sulfate deposition beneath the detached layer (sub-florescence zone), promoting salt crystallization and crust detachments. Though HM limited chloride ingress to some extent (Table 3) compared to uncoated specimens, shrinkage cracks allowed for significant chloride transport through the substrate, resulting in substantial penetration depths (24.5 mm, 28.5 mm, and 30.5 mm for GU, FA, and SG, respectively). Calcium chloride accumulation in surface layers (0–10 mm) induced salt crystallization and progressive peeling.

HM/nanocomposites demonstrated varying degrees of improvement compared to neat HM, depending on exposure type, mixture design, type of nanoparticles, and loading ratio. For PSA conditions, all HM/nanocomposites enhanced concrete resistance, with superior performance observed at the lower loading ratio (2.5%). For instance, the worst-performing mixture (SG) coated with HM/MC2.5 experienced a maximum mass loss of 2.0%, while GU specimens coated with HM/HC2.5 showed no mass loss, maintaining a fully intact surface. Doubling the dosage (5%) decreased resistance and resulted in higher mass losses and visual ratings compared to the 2.5% counterparts due to heterogeneous particle distribution, leading to relatively permeable surfaces. 

**Figure 16:**
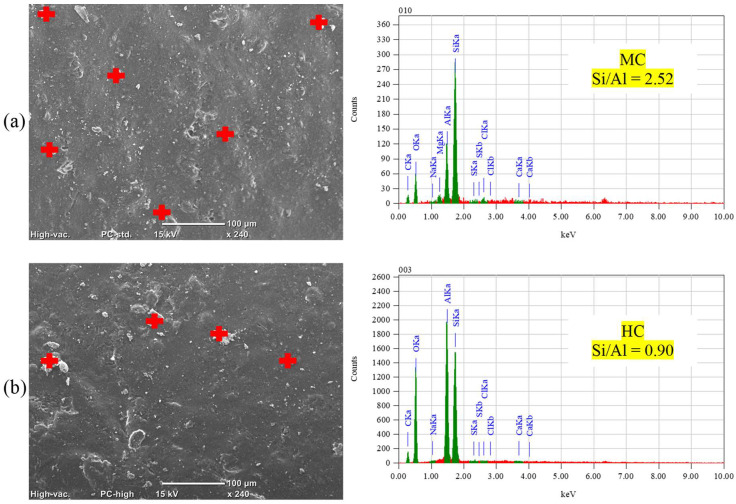
SEM and EDX analyses for surfaces of GU specimens coated with (**a**) HM/MC 2.5 and (**b**) HM/HC 2.5.

**Figure 17:**
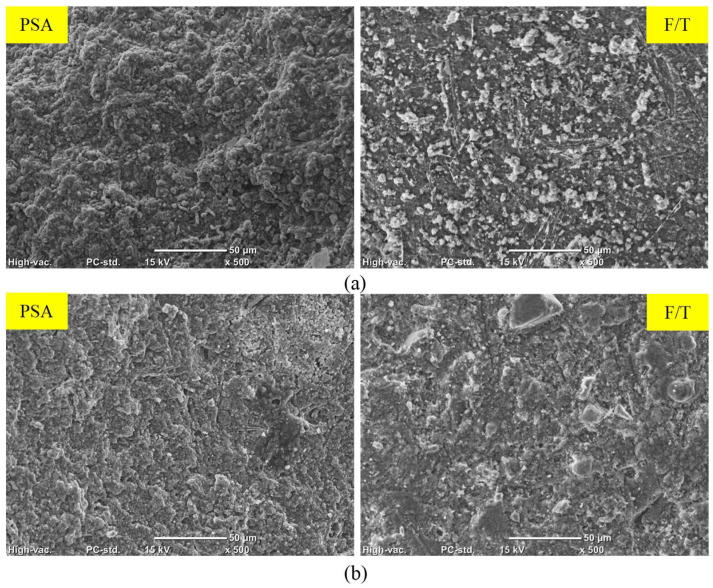
SEM images for SG specimens coated with (**a**) HM/MC 2.5 and (**b**) HM/HC 2.5 after both exposures.

Regarding the type of nanoparticles, similar trends were observed with a slight advantage for HC over MC at equivalent dosages due to its unique shape and lower fineness compared to MC. Figure 1c,d illustrated homogeneous nanocomposites without significant agglomerations for both HM/MC2.5 and HM/HC2.5, indicating successful dispersion that may enhance barrier properties relative to neat HM. Incorporating both nanoparticles with HM significantly reduced shrinkage cracks (Figure 16) and increased bond strength with the concrete surface. These improvements significantly reduced transport indicators (absorption and desorption percentages) for all three mixtures (Figure 2), maintaining nanocomposites on concrete surfaces with minor detachments and notably reducing average mass loss at the end of PSA exposure. HM/MC2.5 and HM/HC2.5 provided superior protection for the weakest mixture (SG), with minimal sodium sulfate presence in the reaction fronts (0 to 10 mm) after 120 cycles (Figure 17), indicating superior protection against severe conditions. 

In the salt–frost scaling exposure, better performances than neat HM were also observed (Figure 6), but only HM/HC2.5 met both MTO and BNQ failure limits across the three mixtures. While HM/MC2.5 reduced chloride penetration depths (Table 3), it did not match the performance of HM/HC2.5 due to more lumps caused by its relatively higher fineness. This led to notable early nanocomposite detachments at these weak locations; upon direct salt exposure, slab surfaces showed performance similar to uncoated slabs at detachment locations. Figure 17a illustrates that SG samples coated with HM/MC 2.5 had a calcium chloride presence in the surface layer, while counterparts coated with HM/HC 2.5 were relatively free of calcium chloride, as shown in Figure 17b.

## 5. Conclusions

Based on the scope of the conducted study, the following conclusions can be drawn:

- High *w*/*b* (0.6) concrete, representative of residential concrete and deteriorating concrete elements, rendered the mixtures extremely vulnerable to harsh exposures, especially when incorporating 40% fly ash and 60% slag.
- The hydrophobic and pore-blocking effects of three layers of ethyl silicate (ES) did not deliver the sufficient protection of concrete under harsh conditions; likewise, high-molecular-weight methyl methacrylate (HM) failed to protect concrete due to shrinkage cracking, rendering it unsuitable for similar exposures in the field.
- The halloysite-based (HC) nanocomposites benefited from better interlocking with the resins due to their unique nano-tube geometry relative to the solid montmorillonite (MC) particles.
- Employing a higher nano-clay dosage (5%), particularly MC, generally led to inferior concrete performance, characterized by increased transport properties and higher mass losses in PSA and salt–frost exposures due to the uneven dispersion of nanocomposites on the concrete surface.
- HM/nanocomposites made with 2.5% HC or MC effectively protected concrete under PSA only (wicking process under the wetting/drying conditions). Conversely, during the salt–frost scaling exposure, the functionality of most HM/nanocomposites were compromised due to continuous ponding of the salt solution with frost cycling, which facilitated their detachment from the surface starting at 15 cycles, especially for the FA and SG specimens.
- Combining 2.5% MC or HC with ES proved effective at safeguarding all tested concretes under both exposures due to the synergistic impact of ES (water-repellent and pore-blocking actions) and optimum dosage of nanoparticles (homogeneous barrier properties), qualifying them as promising candidates for broader field applications. However, field trials are recommended for future research to substantiate these laboratory outcomes.

## Figures and Tables

**Table 1:** Chemical composition and physical properties of binder constituents.

	GU Cement	Fly Ash (FA)	Slag (SG)
**Oxide analysis**			
SiO_2_ (%)	19.2	56.0	33.4
Al_2_O_3_ (%)	5.0	23.1	13.4
Fe_2_O_3_ (%)	2.33	3.6	0.76
CaO (%)	63.2	10.8	42.2
MgO (%)	3.3	1.1	5.3
SO_3_ (%)	3.0	0.2	2.4
Na_2_O_eq._ (%)	0.12	3.2	0.3
**Physical properties**			
Mean particle size, μm	13.15	16.56	14.12
Specific gravity	3.15	2.12	2.87
Fineness, m^2^/kg	390	290	492

**Table 2:** Mixture design of concrete mixtures per cubic meter.

Mixture ID	Cement (kg)	Fly Ash (kg)	Slag (kg)	Water (kg)	Coarse Aggregate (kg)	Fine Aggregate (kg)	56-Day Compressive Strength (MPa)	
							Avg.	SD
**GU**	400	-	-	240	1200	410	35.3	1.53
**FA**	240	160	-	240	1200	346	32.8	1.37
**SG**	160	-	240	240	1200	391	31.4	1.45

Note: SD stand for average values and standard deviation, respectively.

**Table 3:** RCPT results for uncoated and coated specimens at 56 days.

Coating ID	Mixture ID								
	GU			FA			SG		
	Passing Charges (Coulombs)	Penetration Depth (mm)	Migration Coefficient (×10^−12^ m^2^/s)	Passing Charges (Coulombs)	Penetration Depth (mm)	Migration Coefficient (×10^−12^ m^2^/s)	Passing Charges (Coulombs)	Penetration Depth (mm)	Migration Coefficient (×10^−12^ m^2^/s)
**Uncoated**	OVF	50.0	71.7	6480	50.0	47.8	7752	50.0	47.8
**ES**	2773	19.5	18.0	3492	23.0	21.4	4125	28.5	26.7
**ES/MC 2.5**	935	7.5	6.5	1356	10.0	8.9	1864	12.5	11.3
**ES/MC 5**	1643	10.0	8.9	2056	14.0	12.7	2476	18.5	17.0
**ES/HC 2.5**	786	6.0	5.1	967	8.5	7.5	1396	9.5	8.4
**ES/HC 5**	1287	8.5	7.5	1675	12.5	11.3	1976	14.5	13.2
**HM**	3981	24.5	22.8	4826	28.5	26.7	5448	30.5	28.7
**HM/MC 2.5**	840	7.5	6.5	1085	8.5	7.5	1223	10.5	9.4
**HM/MC 5**	2112	12.5	11.3	2795	18.5	17.0	3018	19.0	17.5
**HM/HC 2.5**	797	6.5	5.6	1050	8.0	7.0	1644	11.0	9.8
**HM/HC 5**	1613	11.5	10.3	2254	16.0	14.6	2545	17.5	16.1

Notes: OVF stands for “overflow”, which led to the termination of the RCPT cell before 6 h. The standard deviations of the RCPT results ranged from 7 to 13%.

**Table 4:** Example of analysis of variance (ANOVA) for the results of GU specimens.

Parameter	Migration Coefficient		Absorption		Desorption		PSA Mass Loss		Frost Scaling Mass Loss	
	*F*	*F_cr_*	*F*	*F_cr_*	*F*	*F_cr_*	*F*	*F_cr_*	*F*	*F_cr_*
**Type of Coating**										
Uncoated vs. ES	1227.71 *	18.51	62.68 *	18.51	90.98 *	18.51	48.36 *	7.71	128.33 *	18.51
Uncoated vs. HM	946.63 *	18.51	10.72	18.51	31.45 *	18.51	5.64	7.71	24.55 *	18.51
ES vs. HM	30.37 *	18.51	19.73 *	18.51	27.95 *	18.51	18.50 *	7.71	34.46 *	18.51
**Type of Nanoparticles**										
ES/MC 2.5 vs. ES/HC 2.5	11.46	18.51	36.80 *	18.51	4.73	18.51	31.21 *	7.71	-	18.51
ES/MC 5 vs. ES/HC 5	16.63	18.51	18.15	18.51	35.89 *	18.51	24.90 *	7.71	18.45	18.51
HM/MC 2.5 vs. HM/HC 2.5	13.23	18.51	10.47	18.51	311.44 *	18.51	6.52	7.71	261.36 *	18.51
HM/MC 5 vs. HM/HC 5	4.34	18.51	39.70 *	18.51	43.04 *	18.51	15.84 *	7.71	2.34	18.51
**Dosage**										
ES vs. ES/MC 2.5	330.80 *	18.51	155.05 *	18.51	422.52 *	18.51	101.38 *	7.71	373.26 *	18.51
ES/MC 2.5 vs. ES/MC 5	31.43 *	18.51	25.57 *	18.51	85.77 *	18.51	32.94 *	7.71	121.00 *	18.51
ES vs. ES/HC 2.5	464.39 *	18.51	222.08 *	18.51	588.15 *	18.51	124.15 *	7.71	373.26 *	18.51
ES/HC 2.5 vs. ES/HC 5	47.55 *	18.51	26.90 *	18.51	161.28 *	18.51	82.20 *	7.71	86.22 *	18.51
HM vs. HM/MC 2.5	523.35 *	18.51	189.79 *	18.51	448.98 *	18.51	121.91 *	7.71	194.67 *	18.51
HM/MC 2.5 vs. HM/MC 5	147.45 *	18.51	89.60 *	18.51	13.76	18.51	106.80 *	7.71	52.54 *	18.51
HM vs. HM/HC 2.5	597.42 *	18.51	206.93 *	18.51	864.08 *	18.51	125.14 *	7.71	389.54 *	18.51
HM/HC 2.5 vs. HM/HC 5	180.39 *	18.51	47.81 *	18.51	49.08 *	18.51	175.92 *	7.71	222.40 *	18.51

Notes: * Denotes statistical significance. The *F* and *F_cr_* values are dimensionless.

## Data Availability

Data are contained within the article.

## Appendix A

**Table A1:** Absorption and desorption of specimens.

	Absorption (%)						Desorption (−%)					
	Mixture ID											
Coating ID	GU		FA		SG		GU		FA		SG	
	Avg.	SD	Avg.	SD	Avg.	SD	Avg.	SD	Avg.	SD	Avg.	SD
**Uncoated**	6.80	0.52	7.20	0.61	8.00	0.68	3.16	0.16	3.35	0.19	3.86	0.22
**ES**	3.50	0.28	4.00	0.38	5.10	0.42	1.90	0.09	2.20	0.12	2.60	0.17
**ES/MC 2.5**	0.90	0.08	1.60	0.14	2.50	0.26	0.35	0.05	0.72	0.06	1.53	0.10
**ES/MC 5**	1.30	0.12	2.40	0.23	3.30	0.30	0.90	0.06	1.70	0.11	2.00	0.14
**ES/HC 2.5**	0.45	0.06	0.70	0.08	1.50	0.10	0.26	0.02	0.54	0.05	0.72	0.08
**ES/HC 5**	0.86	0.09	1.20	0.11	2.20	0.23	0.60	0.03	0.82	0.07	1.30	0.09
**HM**	5.20	0.46	5.80	0.51	6.50	0.58	2.40	0.10	2.55	0.12	2.93	0.19
**HM/MC 2.5**	0.68	0.06	1.05	0.14	2.12	0.20	0.83	0.04	0.95	0.05	1.01	0.08
**HM/MC 5**	1.80	0.16	3.00	0.28	3.50	0.31	1.00	0.06	1.82	0.12	2.35	0.15
**HM/HC 2.5**	0.50	0.04	0.82	0.09	1.60	0.17	0.26	0.03	0.50	0.05	0.86	0.08
**HM/HC 5**	1.00	0.09	1.72	0.16	2.80	0.25	0.61	0.06	1.00	0.08	1.72	0.11

Notes: Absorption and desorption values are represented by positive and negative values, respectively. Avg. and SD stand for average values and standard deviation, respectively.

**Table A2:** Variation of mass (%) of uncoated and coated specimens during PSA exposure.

Coating ID	Days/Cycles							
	30		60		90		120	
	Avg.	SD	Avg.	SD	Avg.	SD	Avg.	SD
	**GU**							
**Uncoated**	**−1.61**	**0.28**	**−3.06**	**0.92**	−7.59	1.40	−12.20	1.54
**ES**	−0.36	0.10	−1.26	0.14	−2.88	0.31	−5.20	0.81
**ES/MC 2.5**	+0.10	0.01	+0.17	0.02	−0.24	0.07	−0.44	0.14
**ES/MC 5**	+0.07	0.01	+0.14	0.02	−0.44	0.14	−1.22	0.19
**ES/HC 2.5**	+0.11	0.02	+0.14	0.03	+0.18	0.04	+0.23	0.05
**ES/HC 5**	+0.12	0.03	+0.20	0.02	+0.25	0.03	−0.58	0.11
**HM**	−0.92	0.10	−2.41	0.42	−4.29	0.63	−9.30	1.44
**HM/MC 2.5**	+0.15	0.02	+0.20	0.02	+0.25	0.03	−0.11	0.07
**HM/MC 5**	+0.09	0.01	+0.14	0.02	−0.41	0.08	−1.20	0.17
**HM/HC 2.5**	+0.08	0.03	+0.12	0.02	+0.15	0.03	+0.20	0.05
**HM/HC 5**	+0.11	0.02	+0.14	0.03	+0.20	0.04	−0.75	0.10
	**FA**							
**Uncoated**	−3.60	0.47	−5.87	0.93	−9.64	1.48	−15.80	1.90
**ES**	−0.88	0.20	−2.40	0.30	−3.69	0.44	−6.90	1.26
**ES/MC 2.5**	+0.11	0.03	−0.48	0.12	−0.86	0.16	−1.28	0.40
**ES/MC 5**	+0.09	0.03	−1.23	0.28	−2.19	0.32	−3.14	0.74
**ES/HC 2.5**	+0.14	0.01	+0.18	0.03	+0.22	0.04	−0.36	0.11
**ES/HC 5**	+0.10	0.01	+0.17	0.02	−0.34	0.12	−1.02	0.21
**HM**	−1.73	0.23	−3.40	0.58	−6.30	0.93	−12.70	1.66
**HM/MC 2.5**	+0.16	0.05	+0.18	0.03	+0.26	0.07	−0.56	0.10
**HM/MC 5**	+0.10	0.02	−0.28	0.04	−1.17	0.11	−3.64	0.97
**HM/HC 2.5**	+0.10	0.03	+0.16	0.04	+0.25	0.05	−0.35	0.12
**HM/HC 5**	+0.11	0.03	+0.19	0.05	−0.94	0.25	−1.59	0.35
	**SG**							
**Uncoated**	−5.44	0.72	−9.01	1.15	−14.10	1.72	−20.20	2.06
**ES**	−1.74	0.49	−3.25	0.89	−5.59	1.49	−9.02	1.69
**ES/MC 2.5**	−0.18	0.05	−1.59	0.14	−2.48	0.43	−3.29	0.93
**ES/MC 5**	−0.59	0.15	−2.72	0.63	−3.70	0.38	−6.00	1.08
**ES/HC 2.5**	+0.12	0.03	+0.20	0.04	−0.90	0.30	−1.55	0.39
**ES/HC 5**	+0.16	0.08	−0.40	0.12	−1.48	0.72	−3.05	0.87
**HM**	−2.79	0.43	−5.21	0.72	−8.19	1.48	−17.30	1.86
**HM/MC 2.5**	+0.10	0.01	+0.12	0.04	−0.83	0.20	−2.00	0.54
**HM/MC 5**	+0.14	0.04	−0.40	0.10	−3.08	0.53	−5.61	1.15
**HM/HC 2.5**	+0.12	0.05	+0.21	0.04	−0.59	0.13	−1.30	0.31
**HM/HC 5**	+0.15	0.04	−0.11	0.04	−1.07	0.10	−3.30	0.82

Notes: (−) refers to mass loss and (+) refers to mass gain. Avg. and SD stand for average values and standard deviation, respectively.

**Table A3:** Cumulative scaled mass (g/m^2^) of specimens during salt–frost scaling exposure.

Coating ID	Days/Cycles							
	10		35		35		50	
	Avg.	SD	Avg.	SD	Avg.	SD	Avg.	SD
	**GU**							
**Uncoated**	**264**	**19**	**532**	**24**	**678**	**33**	822	46
**ES**	0	0	48	8	171	18	386	28
**ES/MC 2.5**	0	0	0	0	0	0	0	0
**ES/MC 5**	0	0	0	0	0	0	97	12
**ES/HC 2.5**	0	0	0	0	0	0	0	0
**ES/HC 5**	0	0	0	0	0	0	52	8
**HM**	0	0	78	11	294	19	600	43
**HM/MC 2.5**	0	0	0	0	58	9	155	14
**HM/MC 5**	0	0	29	3	106	10	316	28
**HM/HC 2.5**	0	0	0	0	0	0	0	0
**HM/HC 5**	0	0	0	0	75	9	274	26
	**FA**							
**Uncoated**	351	23	614	39	824	45	1056	59
**ES**	0	0	123	11	296	18	618	40
**ES/MC 2.5**	0	0	0	0	0	0	46	6
**ES/MC 5**	0	0	0	0	125	10	338	29
**ES/HC 2.5**	0	0	0	0	0	0	0	0
**ES/HC 5**	0	0	0	0	85	7	288	23
**HM**	98	4	310	16	631	27	994	35
**HM/MC 2.5**	0	0	67	5	195	11	386	26
**HM/MC 5**	63	6	276	16	542	26	853	52
**HM/HC 2.5**	0	0	0	0	0	0	123	18
**HM/HC 5**	0	0	104	9	276	21	529	33
	**SG**							
**Uncoated**	576	31	995	42	1362	59	1652	89
**ES**	191	13	424	24	688	33	1033	58
**ES/MC 2.5**	0	0	0	0	0	0	71	8
**ES/MC 5**	0	0	109	8	268	19	654	41
**ES/HC 2.5**	0	0	0	0	0	0	0	0]
**ES/HC 5**	0	0	39	3	189	14	557	36
**HM**	136	7	497	20	901	45	1383	64
**HM/MC 2.5**	0	0	114	8	270	21	516	36
**HM/MC 5**	184	13	400	19	767	35	1215	62
**HM/HC 2.5**	0	0	0	0	83	6	245	20
**HM/HC 5**	126	8	322	17	595	29	979	50

Note: Avg. and SD stand for average values and standard deviation, respectively.
